# Susceptibility-guided use of gentamicin-loaded calcium sulfate after surgical debridement for complex diabetes-related foot infection: a single-centre case series

**DOI:** 10.3389/fsurg.2026.1974550

**Published:** 2026-10-07

**Authors:** Omer Arda Cetinkaya, Ilgiz Tuzken, Arda Demirkan, Can Yahya Boztug, Hakan Uncu

**Affiliations:** Department of General Surgery, Ankara University School of Medicine, Ankara, Türkiye

**Keywords:** calcium sulfate, debridement, diabetic foot infection, gentamicin, limb salvage, local antibiotic delivery, wound healing

## Abstract

**Background:**

Complex diabetes-related foot infections (DFIs) frequently involve tissue necrosis, impaired perfusion, bacterial persistence, and residual dead space, which may complicate infection control despite surgical and systemic antimicrobial treatment. Bioabsorbable calcium sulfate provides a potential means of delivering high local antibiotic concentrations after surgical source control. This study describes the clinical, microbiological, wound, and safety outcomes of susceptibility-guided gentamicin-loaded calcium sulfate used as an adjunct to surgical debridement and systemic antimicrobial therapy.

**Methods:**

We retrospectively reviewed 10 consecutive adults with complex DFI treated at a tertiary referral centre between 2024 and 2026. All patients underwent surgical debridement and deep-tissue culture sampling and received systemic antimicrobial therapy. Gentamicin-loaded calcium sulfate was used only when baseline pathogenic isolates demonstrated gentamicin susceptibility. Each application consisted of 5 cc of calcium sulfate mixed with 80 mg gentamicin. Index-wound area, microbiological findings, revascularization, repeat treatment, amputation, laboratory parameters, and safety outcomes were evaluated descriptively.

**Results:**

The mean age was 64.9 years, 90% of patients were male, and peripheral arterial disease was present in 80%. Osteomyelitis was documented in 20%, and 30% underwent lower-extremity revascularization. The median wound-measurement follow-up was 42 days (range, 29–120). Mean index-wound area reduction was 62.9%, and the median reduction was 62.0% (range, 0.6%–100%). Among the nine patients who did not undergo major amputation, all demonstrated reduction in index-wound area; one achieved complete closure during the measurement period and another subsequently underwent definitive closure with a skin graft. One patient required major amputation. Follow-up cultures showed no persistence of baseline pathogens among patients with microbiological reassessment. Median CRP decreased from 66.5 to 9.1 mg/L. No patient developed hypercalcaemia, and no clear cohort-level deterioration in renal function was observed.

**Conclusion:**

In this small uncontrolled case series, susceptibility-guided gentamicin-loaded calcium sulfate was feasible as an adjunct to surgical debridement and systemic antimicrobial therapy in selected patients with complex DFI, with reductions in wound area and absence of baseline-pathogen persistence on available follow-up cultures. However, one patient required major amputation, and the retrospective design, small sample size, multimodal treatment, and absence of a comparator preclude conclusions regarding efficacy. Prospective comparative studies are needed to define the role of this approach in multidisciplinary DFI management.

## Highlights

- Susceptibility-guided gentamicin-loaded calcium sulfate was used as an adjunct to surgical debridement and systemic antimicrobial therapy in 10 patients with complex diabetes-related foot infection.
- Each application consisted of 5 cc calcium sulfate mixed with 80 mg gentamicin, with treatment restricted to infections caused by gentamicin-susceptible isolates.
- Mean index-wound area reduction was 62.9% over a median wound-measurement follow-up of 42 days.
- Follow-up cultures showed no persistence of baseline pathogens among patients with microbiological reassessment.
- One patient required major amputation; no hypercalcaemia or clear cohort-level deterioration in renal function was observed.

## Introduction

Diabetes-related foot infection (DFI) is a complex clinical condition in which microbial burden interacts with tissue necrosis, peripheral neuropathy, mechanical pressure, impaired perfusion, and, in some patients, underlying osteomyelitis. Successful management therefore requires more than antimicrobial therapy alone and typically combines surgical debridement, systemic antibiotics, vascular assessment and revascularization when indicated, pressure offloading, and structured wound care (1–5).

Adequate antimicrobial exposure within an infected diabetic foot wound may be difficult to achieve, particularly in ischemic tissue, residual cavities, and biofilm-containing wound beds. Although an organism may demonstrate antimicrobial susceptibility *in vitro*, impaired tissue perfusion and the presence of biofilm can reduce effective antimicrobial exposure at the site of infection. Bacteria embedded within biofilms may also exhibit substantially greater tolerance to antimicrobial therapy than their planktonic counterparts (6, 7). These factors provide a rationale for investigating local antibiotic-delivery strategies as an adjunct to systemic treatment and surgical source control.

Local antibiotic carriers allow high antimicrobial concentrations to be delivered directly to the debrided wound bed or residual dead space. Importantly, such treatment should not be regarded as a substitute for removal of infected and nonviable tissue. Among available carrier technologies, calcium sulfate has the advantage of being bioabsorbable and therefore does not require subsequent surgical removal, in contrast to non-absorbable polymethylmethacrylate carriers. However, calcium sulfate implantation is not without potential disadvantages; prolonged wound drainage and systemic calcium disturbances have been reported, emphasizing the importance of appropriate patient selection and postoperative surveillance (8, 9).

Evidence supporting local antibiotic delivery in DFI remains limited and heterogeneous. Current IWGDF/IDSA guidance suggests against the routine use of topical or local antibiotics in combination with systemic antimicrobial therapy for diabetes-related soft-tissue infection or osteomyelitis because the available evidence remains of low certainty (1). Nevertheless, several surgical series have reported outcomes with absorbable antibiotic-loaded calcium sulfate or calcium sulfate–hydroxyapatite carriers following debridement of diabetic foot osteomyelitis (10–13). More recently, the randomized BIG D-FOOT study reported fewer early postsurgical infective complications with antibiotic-impregnated calcium sulfate granules, although the trial included only 20 patients before early termination (14).

An important consideration is that local antibiotic delivery should be incorporated into a broader treatment pathway rather than applied indiscriminately to chronic wounds. A potentially rational approach is to combine adequate surgical source control with microbiological confirmation that the selected local antibiotic is active against organisms recovered from deep-tissue cultures. This susceptibility-guided strategy may be particularly relevant in complex wounds characterized by residual dead space, impaired perfusion, or previous failure of conventional wound management.

In contrast to most previous reports, in which antibiotic-loaded calcium sulfate has been used predominantly to fill osseous dead space following resection of infected bone, the present series applied gentamicin-loaded calcium sulfate directly to the debrided soft-tissue wound bed and residual soft-tissue dead space. This soft-tissue application in predominantly non-osteomyelitic complex DFI addresses a distinct and less well-characterized clinical scenario.

The present study therefore evaluated susceptibility-guided gentamicin-loaded calcium sulfate in a selected cohort of patients with complex DFI following surgical debridement. Local gentamicin was used only when baseline deep-tissue pathogens were confirmed to be gentamicin-susceptible and was administered as an adjunct to systemic antimicrobial therapy, vascular optimization when indicated, offloading, and structured wound care. We aimed to describe patient-level microbiological findings, treatment timing, longitudinal index-wound response, vascular interventions, repeat treatment requirements, clinical outcomes, and laboratory safety parameters. Given the small sample size and uncontrolled design, the study was intended to provide hypothesis-generating evidence rather than establish treatment efficacy.

## Materials and methods

### Study design and setting

This retrospective, single-centre case series was conducted in the Department of General Surgery, Ankara University School of Medicine. Consecutive adult patients treated between 2024 and 2026 with gentamicin-loaded calcium sulfate for complex diabetes-related foot infection (DFI) were identified from institutional records. Given the small sample size and absence of a control group, the study was designed as a descriptive, hypothesis-generating surgical case series rather than an efficacy study. The study was conducted in accordance with the Declaration of Helsinki and institutional requirements for retrospective clinical research. All data were analysed in de-identified form.

### Patient selection

Patients were eligible when all of the following criteria were met: age ≥18 years; diabetes mellitus; clinically infected foot ulcer requiring surgical debridement; implantation of gentamicin-loaded calcium sulfate following debridement; an available baseline deep-tissue culture demonstrating susceptibility of the recovered pathogen(s) to gentamicin; and at least one postoperative wound or microbiological assessment.

All patients had undergone conventional wound-care treatment before consideration of local gentamicin-loaded calcium sulfate. Local antibiotic delivery was not used as first-line wound therapy but was considered as an adjunctive treatment in patients with persistent clinical evidence of infection despite previous wound care and systemic antimicrobial therapy, particularly when operative debridement resulted in a residual wound bed or dead space considered at risk for persistent infection.

Patients were excluded when source records were insufficient to define the index wound, baseline microbiological findings, antimicrobial susceptibility, or early clinical outcome. Patients whose baseline deep-tissue isolates were not susceptible to gentamicin were not eligible for local gentamicin-loaded calcium sulfate and were therefore not included.

### Definitions and wound assessment

DFI and infection severity were defined according to IWGDF/IDSA criteria (1). Peripheral arterial disease (PAD) was recorded on the basis of vascular examination, ankle-brachial index, Doppler or duplex ultrasonography, angiographic findings, previous revascularization, or multidisciplinary vascular assessment.

For patients with multiple wounds, an index wound was predefined at the patient level to avoid pseudoreplication. The largest or clinically dominant wound at baseline was selected for longitudinal analysis. Baseline wound area was defined as the wound surface area measured after completion of the index surgical debridement, thereby representing the post-debridement wound bed. Wound surface area was calculated as length  ×  width and expressed in cm^2^. Depth was recorded descriptively when available but was not incorporated into the primary wound-area calculation because depth measurements were not uniformly documented.

Percentage wound-area reduction was calculated as:

[(baseline wound area−final wound area)/baseline wound area] × 100.

Complete index-wound closure was defined as a final documented wound surface area of 0 cm^2^.

### Surgical debridement, microbiological sampling, and local antibiotic application

Each treatment session began with surgical source control. Sharp excisional debridement was performed to remove necrotic, infected, and nonviable soft tissue. Sinus tracts, surrounding callus, and devitalized wound margins were excised until a viable, bleeding wound bed was obtained. Bone was curetted or resected when osteomyelitis was suspected intraoperatively or had been established clinically or radiologically.

Deep-tissue specimens for microbiological culture and antimicrobial susceptibility testing were obtained after debridement and before local antibiotic implantation. Gentamicin-loaded calcium sulfate was used only when the baseline pathogenic organism(s) were confirmed to be susceptible to gentamicin. Antimicrobial susceptibility was interpreted according to European Committee on Antimicrobial Susceptibility Testing (EUCAST) clinical breakpoints; for enterococci, high-level gentamicin susceptibility testing was applied (15).

Following meticulous hemostasis and irrigation with sterile saline, gentamicin-loaded calcium sulfate was prepared intraoperatively using STIMULAN Rapid Cure® (Biocomposites Ltd., Keele, UK). For each application, a 5-cc calcium sulfate kit was mixed with 80 mg gentamicin (2 mL), and the resulting preparation was implanted into the debrided wound bed and/or residual dead space. Local implantation was performed only after adequate surgical source control had been achieved.

When repeat operative treatment was clinically required, surgical debridement was repeated before any subsequent calcium sulfate application. Repeat implantation was not scheduled routinely but was based on subsequent clinical wound assessment and the perceived requirement for renewed source control and further local treatment.

### Systemic antimicrobial therapy

Local gentamicin-loaded calcium sulfate was used as an adjunct to, rather than a replacement for, systemic antimicrobial therapy and surgical source control. Systemic antimicrobial treatment was selected according to the clinical and microbiological findings in consultation with the infectious diseases team.

For the present analysis, the systemic antimicrobial agent and, when available, the interval between initiation of systemic therapy and local calcium sulfate implantation were recorded at the patient level. The interval between baseline deep-tissue culture sampling and local implantation was also recorded to characterize the temporal relationship between microbiological assessment and local treatment.

### Vascular management and offloading

Vascular status was assessed as part of the multidisciplinary treatment pathway using available clinical examination and objective vascular investigations, including ankle-brachial index (ABI), Doppler/duplex ultrasonography, and angiographic assessment, as clinically indicated. Lower-extremity revascularization was performed when arterial perfusion was considered inadequate for wound healing (4). For patients undergoing revascularization, its timing relative to gentamicin-loaded calcium sulfate implantation was recorded. Because of the retrospective design, standardized toe pressure/toe-brachial index (TBI) and transcutaneous oxygen pressure (TcPO₂) measurements were not consistently available.

Postoperative offloading was provided according to individual wound characteristics and clinical requirements. Cast-based total contact offloading was not used in this cohort. Offloading strategies included removable walkers or offloading boots and, where appropriate, custom footwear or heel-relief devices.

### Postoperative wound care

Following implantation, dressing changes were performed twice weekly. At each dressing change, the wound was clinically reassessed and cleaned as required. Residual calcium sulfate material within the wound bed was not routinely mechanically removed and was allowed to remain *in situ* during the resorption period.

Wound appearance and drainage were assessed clinically during routine follow-up. Because exudate volume and its temporal evolution had not been documented using a standardized quantitative or semiquantitative scale, postoperative exudation was not analysed as a formal outcome.

Serial wound dimensions were recorded during follow-up. The change in index-wound surface area between baseline and the last available wound assessment was used to characterize longitudinal wound response.

### Outcomes and safety assessment

The outcomes of interest were microbiological response, change in index-wound surface area, complete index-wound closure, requirement for repeat gentamicin-loaded calcium sulfate application, major amputation, and repeat operative intervention for uncontrolled infection.

Microbiological response was assessed using available follow-up cultures. Follow-up cultures were not obtained according to a predefined research schedule but were collected during clinical follow-up based on the clinical status of the wound. Microbiological response was defined as the absence of persistence of the baseline pathogenic organism(s) in the available follow-up cultures.

Wound response was assessed at the patient level using the predefined index wound and was expressed as percentage change in wound surface area from baseline to the last available measurement.

CRP, white blood cell count, haemoglobin, serum creatinine, blood glucose, and serum calcium were evaluated descriptively using baseline and last available follow-up measurements. Institutional laboratory reference ranges were 0–5 mg/L for CRP, 4.5–11 × 10⁹/L for WBC, 11.7–15.5 g/dL for haemoglobin, 0.5–0.9 mg/dL for serum creatinine, 74–100 mg/dL for glucose, and 8.6–10.2 mg/dL for serum calcium**.** Hypercalcaemia was defined as a serum calcium concentration above the institutional upper reference limit (>10.2 mg/dL).

Because serum gentamicin concentrations were not routinely measured, systemic gentamicin exposure was not directly assessed. Changes in serum creatinine were therefore evaluated descriptively as a renal safety signal rather than as a formal assessment of gentamicin nephrotoxicity.

### Statistical analysis

Because this was an uncontrolled case series of 10 patients, analyses were primarily descriptive and no formal hypothesis testing was performed. Continuous variables were summarized using mean, median, interquartile range (IQR), or range, as appropriate. Categorical variables were reported as number and percentage.

Wound outcomes were analysed at the patient level using the predefined index wound to prevent multiple wounds from the same patient being treated as independent observations. Wound-area change was expressed as both absolute surface area and percentage reduction from baseline. Given the small sample size and descriptive nature of the study, no multivariable modelling or inferential comparative analyses were performed.

## Results

### Patient characteristics

Ten patients with complex diabetes-related foot infection treated with susceptibility-guided gentamicin-loaded calcium sulfate were included. The mean age was 64.9 years, and nine patients (90.0%) were male. Nine patients (90.0%) had type 2 diabetes mellitus and one (10.0%) had type 1 diabetes mellitus. Eight patients (80.0%) were receiving insulin therapy. The median duration of diabetes was 17.5 years (range, 4–35 years)**,** and the mean HbA1c was 8.2%**.**

Peripheral arterial disease was present in eight patients (80.0%), and three patients (30.0%) underwent lower-extremity revascularization during the treatment course. Osteomyelitis was documented in two patients (20.0%). Baseline demographic and clinical characteristics are summarized in Table 1**.**

**Table 1:** Baseline characteristics of the study population (*n* = 10).

Variable	Value
Age, years, mean	64.9
Male sex, *n* (%)	9 (90.0)
BMI, kg/m^2^, mean	26.1
Active smoking, *n* (%)	3 (30.0)
Type 1 diabetes mellitus, *n* (%)	1 (10.0)
Type 2 diabetes mellitus, *n* (%)	9 (90.0)
Diabetes duration, years, median (range)	17.5 (4–35)
Insulin therapy, *n* (%)	8 (80.0)
HbA1c, %, mean	8.2
Peripheral arterial disease, *n* (%)	8 (80.0)
Lower-extremity revascularization, *n* (%)	3 (30.0)
Osteomyelitis, *n* (%)	2 (20.0)

### Microbiological findings and treatment characteristics

Baseline deep-tissue cultures demonstrated a heterogeneous spectrum of pathogens, including *Staphylococcus aureus*, methicillin-resistant *S. aureus* (MRSA), *Staphylococcus lugdunensis*, *Escherichia coli*, *Klebsiella pneumoniae*, *Proteus mirabilis*, *Providencia* spp., *Pseudomonas* spp., and *Enterococcus faecium*. All baseline pathogenic isolates were documented as susceptible to gentamicin before local gentamicin-loaded calcium sulfate was used.

Systemic antimicrobial therapy was administered as part of the multimodal treatment strategy and included clindamycin, ampicillin/sulbactam, meropenem, teicoplanin, and piperacillin/tazobactam. Among patients with reliably documented treatment duration, systemic antimicrobial therapy ranged from 29 to 65 days (Table 2). The duration of oral antimicrobial therapy could not be reliably determined from the retrospective records. One patient received oral clindamycin; in another patient receiving oral systemic antimicrobial therapy, the specific agent was not available in the source records.

**Table 2:** Microbiological findings before and after treatment.

Patient	Baseline deep-tissue culture	Systemic antimicrobial therapy	Culture-to-implantation interval, days	Systemic antimicrobial duration (days)	Revascularization relative to implantation	Repeat application	Follow-up culture
P1	*Staphylococcus aureus*	Clindamycin	5	29	No	No	Skin flora
P2	*Escherichia coli*	Ampicillin/sulbactam	10	43	No	No	Skin flora
P3	*S. aureus* + *S. lugdunensis*	Ampicillin/sulbactam	10	65	14 days after	No	*Corynebacterium*spp. (skin flora)
P4	*E. coli* + *Providencia*spp.	Meropenem	11	60	No	No	No growth
P5	*Proteus mirabilis*	Piperacillin/tazobactam	28	42	49 days before	No	Skin flora
P6	*Klebsiella pneumoniae* + *P. mirabilis* + *Enterococcus faecium*	Meropenem	6	33	No	No	Skin flora
P7	*Pseudomonas* spp.	Oral antimicrobial, agent not documented	8	NR	No	No	Skin flora
P8	MRSA	*Teicoplanin*	3	48	No	No	Skin flora
P9	MRSA	Clindamycin, oral	6	NR	No	No	Skin flora
P10	*E. coli*	Meropenem	7	60	16 days before	Yes, 5 days after first application	Not assessable (major amputation)

Each local application consisted of 5 cc of calcium sulfate mixed with 80 mg gentamicin (2 mL)**.** The interval between baseline deep-tissue culture sampling and implantation ranged from 3 to 28 days. No repeat deep-tissue culture was obtained before implantation in the patient with the longest culture-to-implantation interval of 28 days.

The recorded timing of systemic antimicrobial initiation relative to local implantation varied across patients.

Peripheral arterial disease was present in eight patients. ABI measurements were available in four patients, with recorded values of 1.07, 0.80, 0.92, and 1.08; the ABI of 1.08 was recorded in a patient without PAD. In several patients with PAD, ankle arteries were documented as non-compressible, precluding reliable ABI measurement. Standardized toe pressure/TBI and TcPO₂ measurements were not consistently available in the retrospective records. Three patients underwent lower-extremity revascularization. Two procedures were performed before calcium sulfate implantation, 49 and 16 days before implantation, respectively, whereas one was performed 14 days after implantation. Among the remaining patients with PAD, the specific reasons for not performing revascularization could not be reliably determined from the retrospective records. One patient (10.0%) underwent repeat surgical debridement and a second gentamicin-loaded calcium sulfate implantation 5 days after the initial application. Patient-level microbiological findings, systemic antimicrobial therapy, treatment timing, revascularization, and repeat application are detailed in Table 2.

Follow-up cultures were available in nine of the 10 patients (all except the patient who underwent major amputation) and showed no persistence of the baseline pathogenic organisms. Follow-up specimens predominantly yielded skin flora; one yielded *Corynebacterium* spp. interpreted as skin flora, and one showed no microbial growth.

### Wound and clinical outcomes

The median interval from calcium sulfate implantation to the final index-wound measurement was 42 days (range, 29–120 days)**.** The mean reduction in index-wound surface area was 62.9%**,** and the median reduction was 62.0% (range, 0.6%–100%)**.**

Among the nine patients who did not undergo major amputation, all demonstrated a reduction in index-wound surface area during follow-up. One patient achieved complete index-wound closure during the wound-measurement period, corresponding to a 100% reduction in surface area (Figure 1). Another patient demonstrated a 46.0% reduction during the measured follow-up period and subsequently underwent definitive wound closure with a skin graft. Marked wound-area reductions were also observed in several other patients, including reductions of 98.7%, 88.8%, 88.6%, and 78.0% (Figure 2).

**Figure 1:**
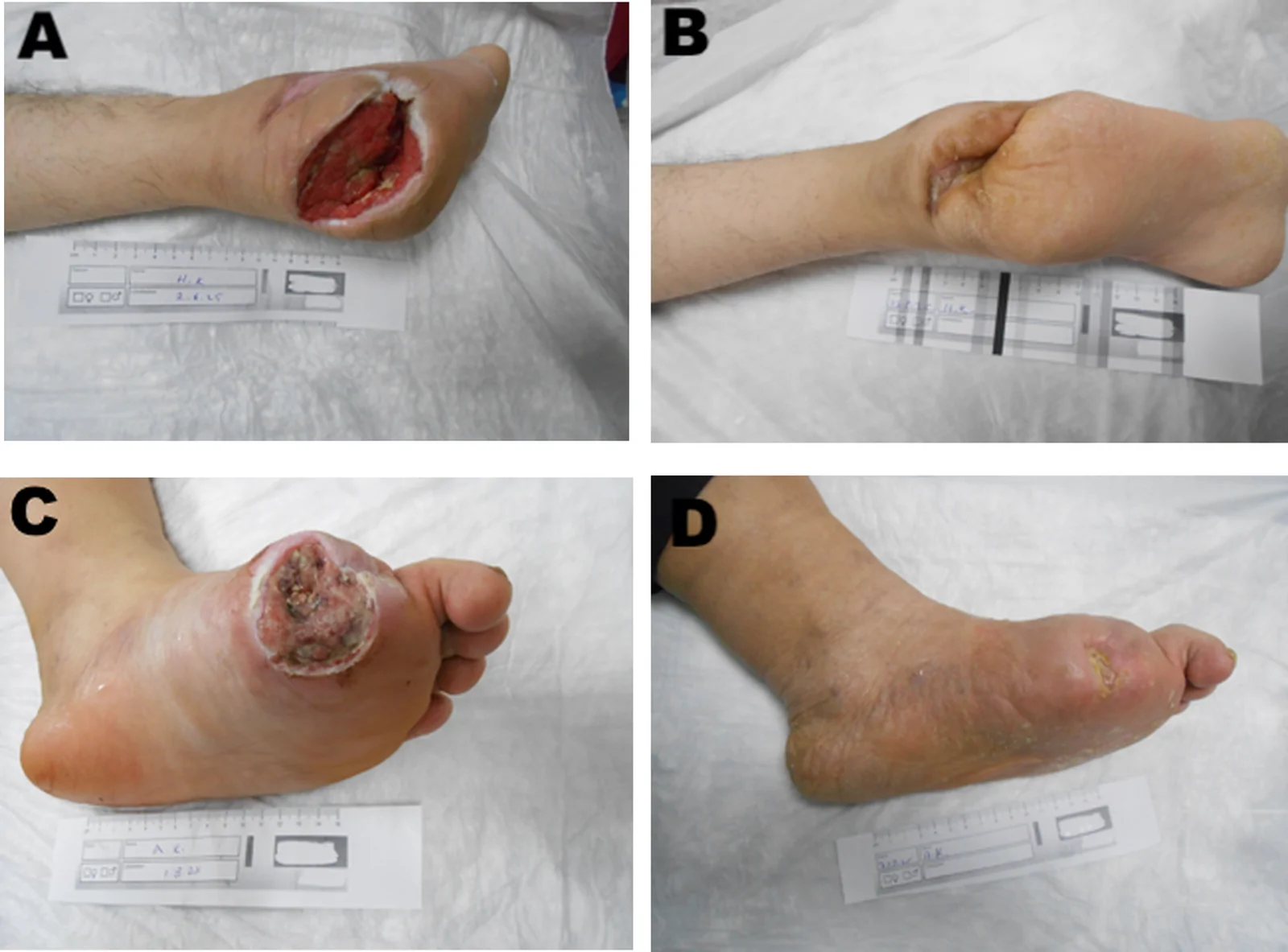
Representative clinical outcomes after debridement and local gentamicin-loaded calcium sulfate. **(A)** Baseline heel diabetes-related foot infection. **(B)** Complete heel wound healing during follow-up. **(C)** Baseline forefoot infection. **(D)** Marked wound reduction and infection control after treatment.

**Figure 2:**
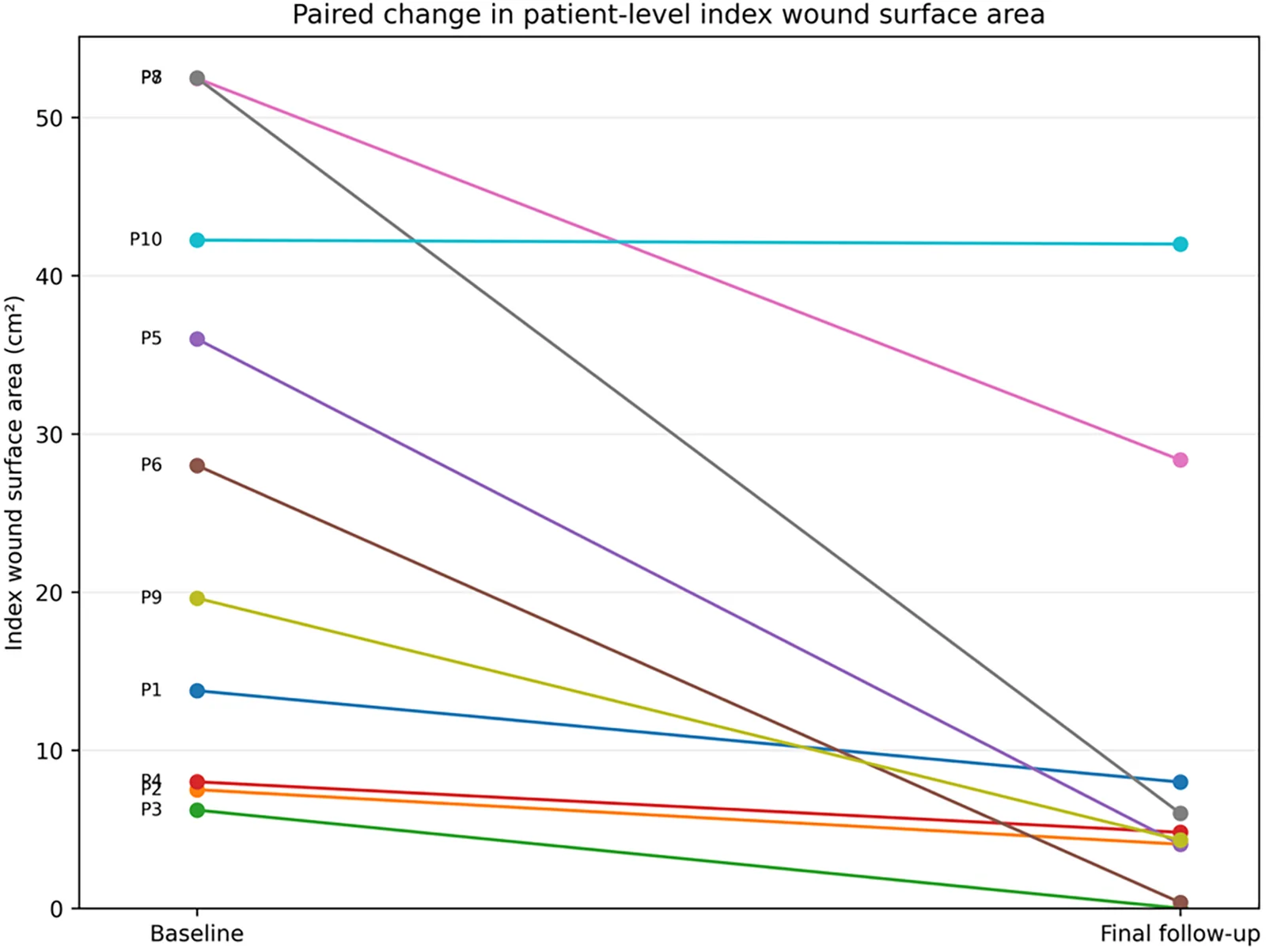
Paired change in patient-level index wound surface area. Wound area was calculated as length  ×  width; completely closed wounds were assigned a final area of 0 cm^2^. Mean index-wound area reduction was 62.9%, and median reduction was 62.0% (range, 0.6%–100%). One patient demonstrated minimal wound-area reduction before subsequently undergoing major amputation.

One patient demonstrated minimal change in index-wound area before ultimately undergoing major amputation; this numerical change was not considered a favourable wound response. Overall, major amputation occurred in one patient (10.0%)**,** whereas no minor amputations were recorded. Individual index-wound trajectories and clinical outcomes are presented in Table 3**.**

**Table 3:** Patient-level index wound outcomes.

Patient	Baseline index-wound area (cm^2^)	Final index-wound area (cm^2^)	Wound-area reduction (%)	Measurement interval (days)	Clinical outcome
P1	13.76	7.98	42.0	39	Wound area reduced
P2	7.50	4.05	46.0	29	Wound area reduced
P3	6.21	0	100.0	91	Complete index-wound closure
P4	8.00	4.80	40.0	43	Wound area reduced
P5	36.00	4.05	88.8	120	Wound area reduced
P6	28.00	0.36	98.7	84	Wound area reduced
P7	52.48	28.35	46.0	31	Subsequent closure with skin graft
P8	52.50	6.00	88.6	64	Wound area reduced
P9	19.61	4.32	78.0	41	Wound area reduced
P10	42.25	42.00	0.6	39	Major amputation

No deaths occurred during the follow-up period.

### Laboratory and safety outcomes

Median CRP decreased from 66.5 mg/L (IQR, 6.9–159.5) at baseline to 9.1 mg/L (IQR, 5.3–15.0) at follow-up. Median WBC count decreased from 8.37 × 10⁹/L (IQR, 6.75–10.79) to 7.85 × 10⁹/L (IQR, 7.26–9.46).

Median haemoglobin increased from 10.35 g/dL (IQR, 10.20–11.50) to 10.95 g/dL (IQR, 10.25–11.78), while median blood glucose decreased from 151 mg/dL (IQR, 125–195.5) to 125 mg/dL (IQR, 100–177.5).

Median serum creatinine was 1.23 mg/dL (IQR, 1.04–1.58) at baseline and 1.15 mg/dL (IQR, 0.94–1.30) at follow-up. Although creatinine concentrations were above the institutional reference range (0.5–0.9 mg/dL) in several patients, including patients with pre-existing chronic kidney disease, no clear cohort-level deterioration in renal function was observed during follow-up.

Median serum calcium was 8.80 mg/dL (IQR, 8.73–8.98) at baseline and 9.10 mg/dL (IQR, 8.78–9.18) at follow-up. No patient developed hypercalcaemia during follow-up (institutional reference range, 8.6–10.2 mg/dL). Laboratory findings are summarized in Table 4.

**Table 4:** Laboratory and safety endpoints.

Laboratory parameter	Institutional reference range	Baseline, median (IQR)	Follow-up, median (IQR)
CRP, mg/L	0–5	66.5 (6.9–159.5)	9.1 (5.3–15.0)
WBC, × 10⁹/L	4.5–11	8.37 (6.75–10.79)	7.85 (7.26–9.46)
Haemoglobin, g/dL	11.7–15.5	10.35 (10.20–11.50)	10.95 (10.25–11.78)
Creatinine, mg/dL	0.5–0.9	1.23 (1.04–1.58)	1.15 (0.94–1.30)
Glucose, mg/dL	74–100	151 (125–195.5)	125 (100–177.5)
Calcium, mg/dL	8.6–10.2	8.80 (8.73–8.98)	9.10 (8.78–9.18)

CRP, C-reactive protein; WBC, white blood cell count; IQR, interquartile range. Laboratory parameters are presented descriptively; no inferential comparison was performed because of the small sample size and uncontrolled case-series design.

## Discussion

In this retrospective single-centre case series of 10 patients with complex diabetes-related foot infection, susceptibility-guided gentamicin-loaded calcium sulfate was used as an adjunct to surgical debridement, systemic antimicrobial therapy, vascular optimization when indicated, offloading, and structured wound care. During a median wound-measurement follow-up of 42 days, the mean index-wound area reduction was 62.9% and the median reduction was 62.0%. Among the nine patients who did not undergo major amputation, all demonstrated reduction in index-wound surface area; one achieved complete closure during the measurement period, and another subsequently underwent definitive closure with a skin graft. Conversely, one patient showed minimal wound-area improvement and ultimately required major amputation. Follow-up cultures showed no persistence of the baseline pathogenic organisms among patients with microbiological reassessment. These findings should be interpreted as descriptive observations within a multimodal treatment pathway rather than evidence of an independent treatment effect of local gentamicin delivery.

The surgical context in which local antibiotic therapy was used is central to interpreting these findings. Diabetes-related foot infection frequently coexists with tissue necrosis, ischemia, neuropathy, mechanical loading, and impaired wound healing, and successful management therefore requires more than antimicrobial therapy alone (1–5). In the present cohort, every calcium sulfate implantation was preceded by surgical debridement with removal of necrotic and nonviable tissue. Local gentamicin was therefore not intended to sterilize untreated necrotic tissue or replace surgical source control. Similarly, all patients received systemic antimicrobial therapy as part of their treatment pathway. The wound and microbiological outcomes observed in this series cannot therefore be attributed to the calcium sulfate carrier alone.

Local antibiotic delivery may offer a theoretical advantage after debridement by achieving high antimicrobial concentrations within residual wound cavities and dead space. This may be particularly relevant in biofilm-containing environments, in which bacteria can exhibit substantially greater antimicrobial tolerance than their planktonic counterparts (6, 7). Calcium sulfate is bioabsorbable and, unlike non-absorbable polymethylmethacrylate carriers, does not routinely require a subsequent operation solely for carrier removal. Previous studies have described the use of antibiotic-loaded calcium sulfate or calcium sulfate–hydroxyapatite carriers following surgical treatment of diabetic foot osteomyelitis and other complex bone infections (8, 10–13, 16).

An important feature of the present series was the microbiologically selected use of local gentamicin. Gentamicin-loaded calcium sulfate was implanted only when organisms recovered from baseline deep-tissue cultures were documented as susceptible to gentamicin. This differs from empirical local antibiotic application and provides microbiological concordance between the implanted antimicrobial and the organisms identified in the wound. Baseline cultures in our cohort were heterogeneous and included Gram-positive and Gram-negative organisms, reflecting the microbiological complexity of DFI. Follow-up cultures showed no persistence of baseline pathogens among patients who underwent microbiological reassessment. However, this finding should not be interpreted as evidence that local gentamicin alone eradicated infection, because surgical debridement and systemic antimicrobial therapy were administered concomitantly and follow-up cultures were obtained selectively according to the clinical status of the wound rather than according to a standardized research schedule. In addition, the interval between baseline culture sampling and local implantation varied across patients and reached 28 days in one patient, without repeat deep-tissue culture before implantation. Because systemic antimicrobial therapy was administered during the treatment pathway, changes in the wound microbiological profile during this interval cannot be excluded. Therefore, baseline susceptibility results may not necessarily reflect the microbiological status of the wound at the time of implantation in patients with longer culture-to-implantation intervals.

The available evidence for local antibiotic delivery in DFI remains limited and heterogeneous. Qin et al. reported improved outcomes when antibiotic-impregnated calcium sulfate was added following infected bone resection in diabetic forefoot osteomyelitis (10), while other retrospective experiences have reported less consistent outcomes (11–13). More recently, the randomized BIG D-FOOT study reported fewer early postsurgical infective complications with antibiotic-impregnated calcium sulfate granules; however, the trial included only 20 patients before early termination (14). Differences in infection phenotype, carrier composition, antimicrobial selection, surgical technique, vascular status, and outcome definitions make direct comparison between studies difficult. Consistent with this uncertainty, current IWGDF/IDSA guidance suggests against routine use of topical or local antibiotics in combination with systemic antimicrobial therapy for diabetes-related soft-tissue infection or osteomyelitis because the available evidence remains of low certainty (1). Our findings therefore should not be interpreted as supporting routine calcium sulfate implantation in DFI.

A potentially relevant distinction between the present cohort and much of the published literature is the indication for local carrier implantation. Previous reports have predominantly focused on diabetic foot osteomyelitis and filling of osseous dead space after infected bone resection (10–14). In contrast, only two patients in our cohort had documented osteomyelitis, and gentamicin-loaded calcium sulfate was used predominantly within the debrided soft-tissue wound bed or residual soft-tissue dead space. The present observations therefore describe a somewhat different clinical setting, although the small uncontrolled design prevents any conclusions regarding comparative effectiveness.

The wound response was heterogeneous and should be interpreted separately from microbiological control. Although the mean index-wound area reduction was 62.9%, complete closure during the measured follow-up period occurred in only one patient, and another patient subsequently required skin grafting for definitive closure. Most importantly, one patient demonstrated minimal wound-area improvement and ultimately underwent major amputation. Antimicrobial-resistant infections may further complicate diabetic foot management and contribute to amputation and subsequent long-term functional disability (17).

This highlights the distinction between reduction in measured wound surface area and clinically meaningful endpoints such as definitive closure and limb salvage. Wound-area reduction should therefore be considered an intermediate outcome rather than evidence of treatment success.

Peripheral arterial disease was present in eight of 10 patients, and three patients underwent lower-extremity revascularization. Two procedures were performed before local calcium sulfate implantation and one after implantation. Perfusion is a major determinant of both infection control and wound healing in diabetic foot disease, and vascular assessment and revascularization when indicated are integral components of contemporary management (4). Consequently, vascular intervention represents an important potential contributor to the observed wound trajectories and further limits any attempt to isolate the independent effect of local gentamicin.

Repeat implantation was uncommon. Only one patient underwent a second debridement and repeat calcium sulfate implantation, 5 days after the initial procedure, and this patient ultimately required major amputation. The present dataset is therefore insufficient to determine whether or when repeat implantation may be beneficial. Rather than supporting scheduled repeat application, these observations suggest that additional debridement and local treatment should remain individualized according to ongoing clinical evidence of inadequate source control.

Safety is an important consideration when absorbable calcium sulfate carriers are used. Prolonged wound drainage has been reported following calcium sulfate implantation, and hypercalcaemia has also been described, particularly in settings involving larger implanted volumes (8, 9). In the present series, each application used a relatively small standardized carrier volume of 5 cc. Median serum calcium remained within the institutional reference range, changing from 8.80 mg/dL at baseline to 9.10 mg/dL at follow-up, and no patient developed hypercalcaemia. Nevertheless, the small sample size limits the ability of this study to establish the safety of calcium sulfate implantation more broadly.

Renal function was also evaluated descriptively because gentamicin was locally administered. Median serum creatinine was 1.23 mg/dL at baseline and 1.15 mg/dL at follow-up. Several patients had creatinine values above the institutional reference range, including patients with pre-existing chronic kidney disease, but no clear cohort-level deterioration was observed. However, serum gentamicin concentrations were not measured, and the study was not designed to quantify systemic gentamicin exposure. The creatinine findings should therefore be regarded as descriptive safety observations rather than evidence excluding systemic toxicity.

Inflammatory markers showed a favourable descriptive trend, particularly CRP, which decreased from a median of 66.5 mg/L at baseline to 9.1 mg/L at follow-up. WBC values also remained relatively stable. These changes are compatible with improvement in overall infection burden but are nonspecific and may reflect the combined effects of debridement, systemic antimicrobial treatment, vascular optimization, and continued wound care. No inferential testing was performed because of the small sample size and descriptive study design.

Several limitations should be acknowledged. First, this was a retrospective, single-centre case series involving only 10 patients without a comparator group, precluding causal inference or estimation of treatment efficacy. Second, treatment was individualized according to clinical requirements, creating potential selection bias and confounding by indication. Third, the cohort was heterogeneous with respect to vascular status, microbiological findings, systemic antimicrobial regimens, wound characteristics, and subsequent reconstructive management. In addition, objective limb-perfusion assessment was not standardized. ABI could not be reliably obtained in several patients because of non-compressible ankle arteries, and toe pressure/TBI and TcPO₂ measurements were not consistently available. Furthermore, the retrospective records did not permit uniform determination of the clinical rationale for revascularization versus conservative vascular management in all patients with PAD. Consequently, the contributions of baseline limb perfusion and revascularization to wound-healing outcomes cannot be separated from those of surgical debridement, systemic antimicrobial therapy, and local antibiotic treatment. Fourth, follow-up duration was relatively short and variable, and wound measurements were obtained during routine clinical care rather than at standardized research time points. Fifth, wound surface area was calculated using two-dimensional length  ×  width measurements because depth and wound volume were not consistently available. In addition, because baseline wound area was recorded after surgical debridement, which necessarily enlarges the wound, the observed reductions may partly reflect resolution of the post-debridement surgical wound rather than the effect of local antibiotic delivery alone. Sixth, follow-up microbiological sampling was not performed at standardized intervals. Seventh, postoperative exudation was not systematically quantified, preventing formal assessment of calcium sulfate-associated drainage. Finally, serum gentamicin concentrations were unavailable, precluding direct assessment of systemic antimicrobial exposure.

Despite these limitations, this series provides detailed patient-level information regarding microbiological selection, culture-to-implantation timing, concomitant systemic antimicrobial treatment, standardized local gentamicin dose and calcium sulfate volume, vascular intervention, repeat implantation, wound trajectories, and laboratory safety parameters. These data provide a pragmatic description of susceptibility-guided local antibiotic delivery within multidisciplinary management of complex DFI and support further prospective evaluation using standardized indications, wound and microbiological assessments, longer follow-up, systematic safety monitoring, and an appropriate comparator group.

## Conclusion

In this small, uncontrolled case series, susceptibility-guided gentamicin-loaded calcium sulfate was feasible as an adjunct to surgical debridement and systemic antimicrobial therapy in selected patients with complex diabetes-related foot infection. Most patients demonstrated substantial reduction in index-wound area, and baseline pathogenic organisms did not persist in available follow-up cultures. However, one patient ultimately required major amputation.

No hypercalcaemia was observed, and no clear cohort-level deterioration in renal function was identified during follow-up. Given the retrospective design, small sample size, heterogeneous multimodal treatment, and absence of a comparator group, these findings should not be interpreted as evidence of efficacy attributable to local gentamicin delivery. Prospective comparative studies with standardized wound, microbiological, vascular, and safety assessments are needed to define the role of susceptibility-guided local antibiotic delivery in the multidisciplinary management of complex DFI.

## Data Availability

The raw data supporting the conclusions of this article will be made available by the authors, without undue reservation.
